# Gallbladder adenosquamous carcinoma: a case report and literature review

**DOI:** 10.1093/jscr/rjab365

**Published:** 2021-09-27

**Authors:** Tyler Davis, Paige Moudy, Mohamed Abdelgawad, Lutfi Barghuthi, Hishaam Ismael

**Affiliations:** University of Texas Health Science Center at Tyler, Tyler, TX, USA; Texas College of Osteopathic Medicine, Fort Worth, TX, USA; University of Texas Health Science Center at Tyler, Tyler, TX, USA; University of Texas Health Science Center at Tyler, Tyler, TX, USA; University of Texas Health Science Center at Tyler, Tyler, TX, USA

## Abstract

Representing 90–95% of all malignant gallbladder neoplasms, adenocarcinoma is by far the most common subtype. Adenosquamous carcinoma is a rare subtype, accounting for only 1–5% of all gallbladder carcinomas. These tumors have been shown to have aggressive biologic behavior, commonly extending to adjacent structures. Some studies have shown that the squamous component often displayed a greater proliferative capacity than the adenocarcinomatous component (possibly even up to twice as fast). Complete surgical resection is currently the mainstay of treatment but the prognosis is often poor. In this paper, we present a case of a 69-year-old male with an AJCC Stage IV moderately differentiated adenosquamous carcinoma of the gallbladder treated with radical cholecystectomy including liver segments IVb, V, VI.

## INTRODUCTION

Although rare, gallbladder carcinoma is the most common type of biliary tract cancer. It has a clear predominance for females, but its geographical prevalence is widely variable, suggesting a complex association with genetic and environmental factors. Risk factors include cholelithiasis, obesity, gallbladder polyps and female sex. Many histologic subtypes exist. Representing 90–95% of all malignant gallbladder neoplasms, adenocarcinoma is by far the most common subtype. Adenosquamous carcinoma is a rare subtype, accounting for only 1–5% of all gallbladder carcinomas. Strong controversy exists over its histogenesis. Some believe that it is squamous differentiation in an adenocarcinoma, while others think it may be closely related to the neoplastic process of squamous cell carcinoma. Regardless of how they arise, these tumors have been shown to have aggressive biologic behavior, commonly extending to adjacent structures including the liver, omentum, stomach, duodenum and transverse colon. Some studies have shown that the squamous component often displayed a greater proliferative capacity than the adenocarcinomatous component. Complete surgical resection is currently the mainstay of treatment, but the prognosis is often poor.

In this paper, we present a case of a 69-year-old male with an AJCC Stage IV moderately differentiated adenosquamous carcinoma of the gallbladder treated with radical cholecystectomy including liver segments IVb, V, VI.

## CASE PRESENTATION

This paper presents a 69-year-old male initially presenting to the clinic for a new liver lesion identified on screening CT scan. Review of systems was significant for fatigue, weight loss, easy bruising/bleeding, chronic back and joint pain. The patient’s past medical history is significant for tobacco use, cholelithiasis, left renal cell carcinoma status-post left robotic-assisted partial nephrectomy, bilateral Warthrin’s Tumor status-post bilateral superficial parotidectomy, benign prostatic hyperplasia, hypertension and coronary artery disease.

**Figure 1:**
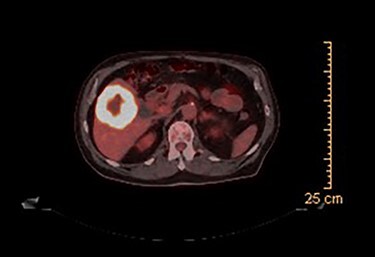
PET scan (axial view): hypermetabolic, centrally necrotic mass involving liver segment V, VI.

**Figure 2:**
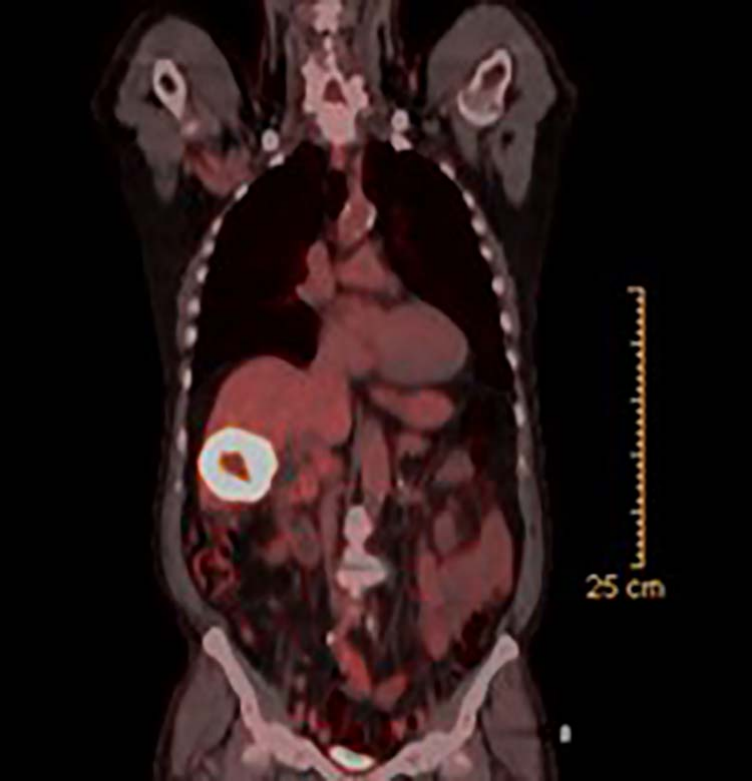
PET scan (coronal view).

CT guided biopsy of the lesion demonstrated the presence of invasive squamous cell carcinoma with p40-positive and focally positive for CK7 cells. Caris testing was positive for PDL1 (95%) with stable MMR and low TMB. PET scan revealed a large hypermetabolic, centrally necrotic mass involving liver segments V, VI without any abnormal FDG uptake within the head, neck or chest. Pertinent images from the PET scan are demonstrated in Figs 1 and 2.

**Figure 3:**
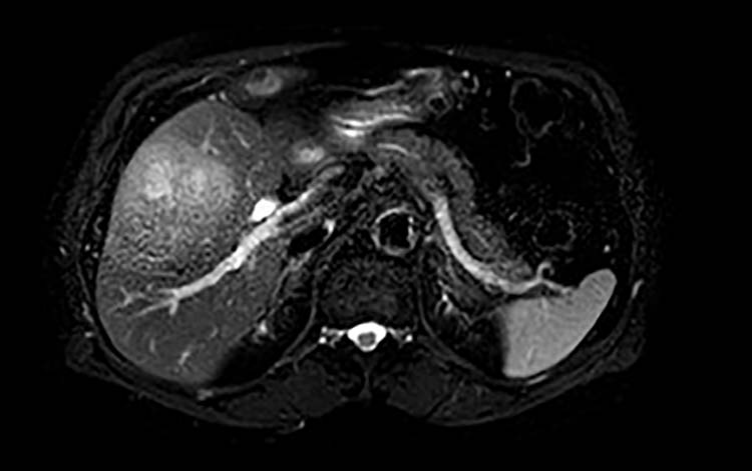
MRI (axial view): 7.1 × 8.1 cm mass originating from the gallbladder involving segments IVb, V, VI with areas of peripheral enhancement and central necrosis.

**Figure 4:**
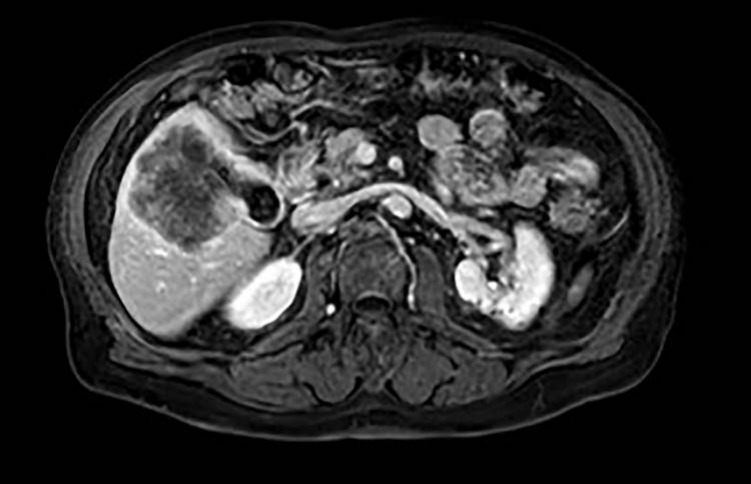
MRI (axial view).

**Figure 5:**
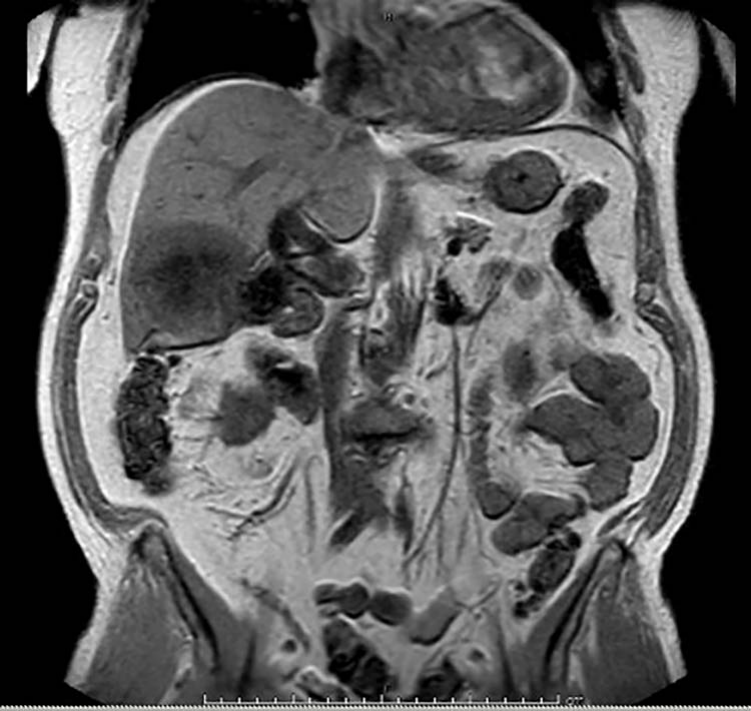
MRI (coronal view).

MRI demonstrated a 7.1 × 8.1 cm mass originating from the gallbladder that involved segments IVb, V, VI with areas of peripheral enhancement and central necrosis. Associated cholelithiasis was also noted. Figures 3–5 demonstrate the pertinent MRI findings.

This patient was treated with a radical cholecystectomy including liver segments IVb, V, VI and portal lymphadenectomy. Using a modified Makuuchi incision, the liver, colon and duodenum were mobilized. There was no identifiable tumor involvement of the bowel. The cystic duct was isolated and divided at the bile duct. A frozen section of the cystic duct was sent for analysis which revealed negative margins. Portal lymphadenectomy and resection of liver segments IVb, V, VI en-bloc with the gallbladder was performed and sent for histopathologic analysis. Falciform and omental pedicle flaps were created and two drains were placed. The patient tolerated the procedure well with no acute intraoperative complications. His post-operative course was unremarkable and he was discharged on post-operative Day 4.

**Figure 6:**
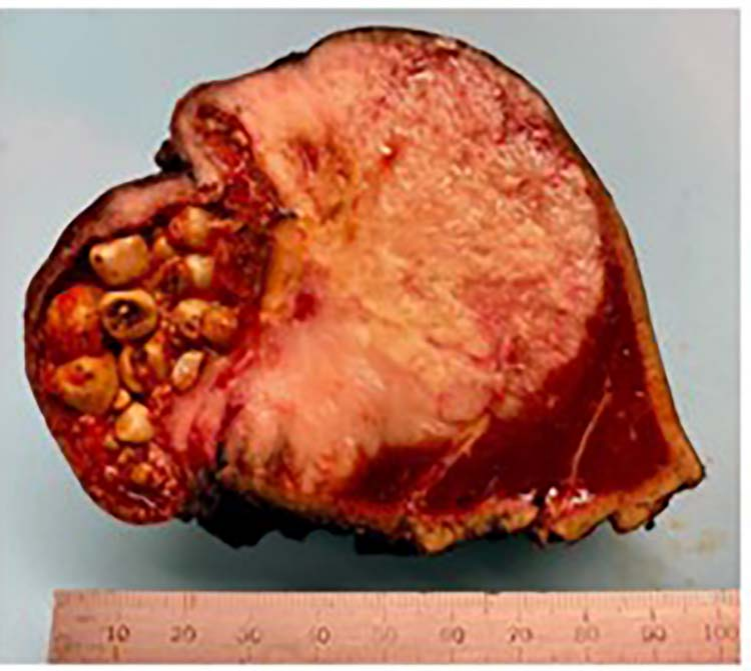
Gross cross section of the gallbladder demonstrating tumor involvement and cholelithiasis.

**Figure 7:**
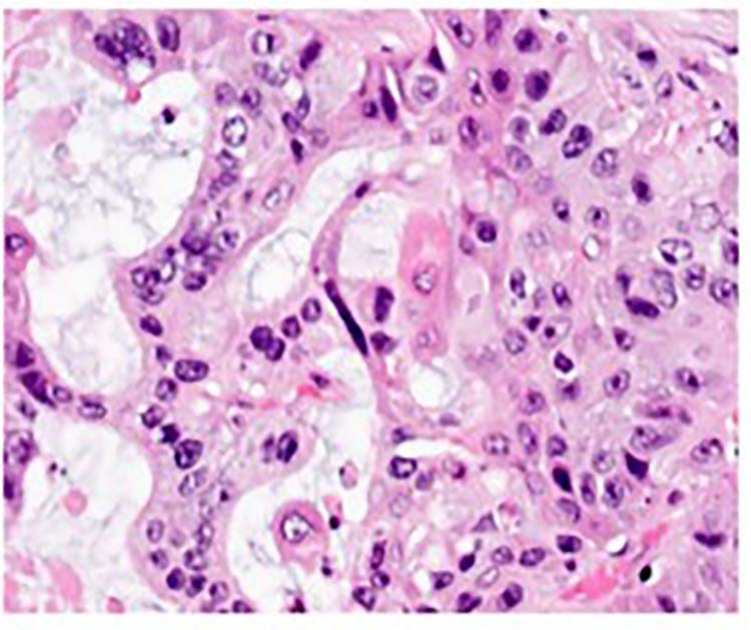
H&E stain of the tumor demonstrating adenosquamous features.

**Figure 8:**
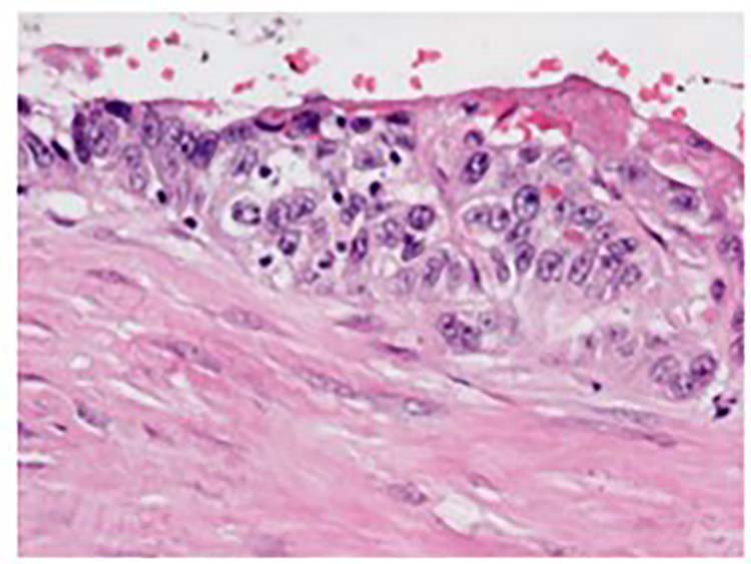
H&E stain demonstrating peritoneal surface involvement.

**Figure 9:**
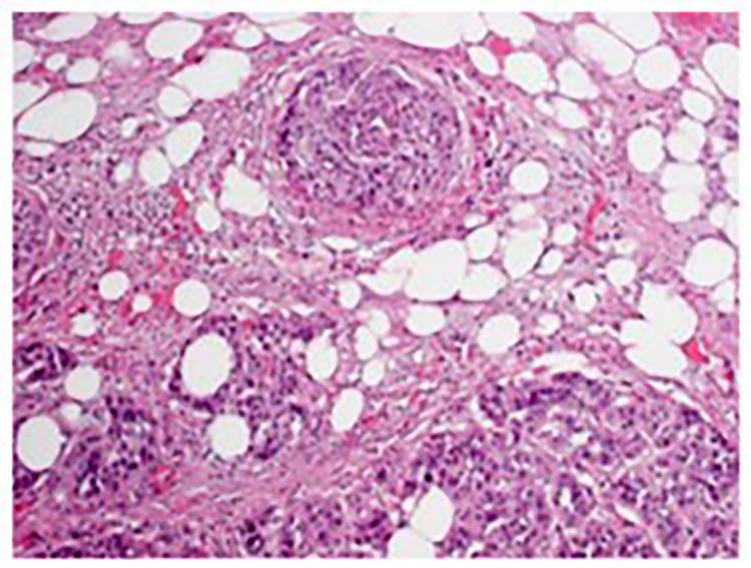
H&E stain demonstrating involvement of the lesser omentum.

Pathologic analysis revealed an AJCC Stage IV (pT3, N0, M1) 7.7 cm moderately differentiated (histologic grade G2) adenosquamous carcinoma of the gallbladder with lymphovascular and perineural invasion, and tumor extension into adjacent liver, peritoneal surface and residual lesser omentum. Surgical margins were all negative. No tumor was identified in any of the eight portal lymph nodes obtained. Figure 6 shows a gross cross section of the gallbladder. Figure 7 shows H&E stain of the tumor demonstrating adenosquamous features. Figure 8 shows an H&E stain demonstrating peritoneal surface involvement. Figure 9 shows an H&E stain demonstrating involvement of the lesser omentum.

## DISCUSSION

Gallbladder adenosquamous carcinomas are a rare occurrence. Literature review of 69 cases (summarized in Table 1) demonstrated that the average patient age to be 66.6 years old ranging from 43 to 89 years old [1–12]. There was a clear gender predominance with 72.5% (50/69) patients being female, yielding a Female: Male Ratio of 2.6:1. This statistic is similar to those previously reported in the literature. Cholelithiasis was reported in over half of these patients, again suggesting its association as a risk factor for developing gallbladder carcinoma.

**Table 1:** Literature review of 69 cases of gallbladder adenosquamous carcinoma

**Article title**	**Author(s)**	**Age**	**Gender**	**Presentation**	**Tumor Size**	**Radical resection performed?**	**Primary tumor resection performed?**	**T**	**N**	**M**	**Stage**	**Involvement of adjacent structures**	**Outcome (months, status)**
Adenosquamous carcinoma of the gallbladder warrants resection only if curative resection is feasible	Oohashi Y, Shirai Y, Wakai T, Nagakura S, Watanabe H, Hatakeyama K.	47	M	Cholelithiasis in 15 patients	Mean: 7 cm Range: 2.5–14 cm	X		4	2a	0	IVb	Liver	154, Alive
		65	F			X		4	1a	0	IVa	Liver, omentum	151, Alive
		52	F			X		3	0a	0	III	Liver	Alive
		60	F			X		1a	0a	0	I	None	121, Alive
		69	F			X		4	1a	0	IVa	Liver, colon, duodenum	62, Alive
		62	M			X		4	2a	0	IVb	Liver, colon, duodenum, pancreas	48, Alive
		66	F			X		2	0a	0	III	None	156, Died of other causes
		84	F			X		3	2a	1	IVb	Bile duct	23, Died of Disease
		70	F			X		3	2a	1	IVb	Omentum	13, Died of Disease
		64	F			X		4	2a	1	IVb	Liver, duodenum, stomach	6, Died of other causes
		77	F			X		4	2a	0	IVb	Liver	5, Died of other causes
		62	F			X		4	2a	0	IVb	Liver	5, Died of Disease
		70	M			X		3	0a	0	III	Liver	4, Died of Disease
		83	F			X		4	0a	0	IVa	Liver, colon	4, Died of Disease
		70	F			X		4	0a	0	IVa	Liver, omentum	3, Died of Disease
		78	M				X	2	0b	0	II	None	45, Died of other causes
		60	F				X	2	2b	0	IVb	None	19, Died of Disease
		74	F				X	3	2c	1	IVb	Liver	8, Died of other causes
		72	F				X	2	2b	0	IVb	None	7, Died of other causes
		43	F				X	4	1b	0	IVa	Liver, colon	5, Died of other causes
		62	M				X	4	2b	1	IVb	Liver, colon	5, Died of other causes
		46	F				X	3	2b	1	IVb	Liver	4, Died of other causes
		67	F				X	3	2c	0	IVb	Liver	3, Died of other causes
		78	F				X	2	2c	0	IVb	None	3, Died of Disease
		75	F				X	3	0c	0	III	None	3, Died of Disease
		56	M				X	4	2c	0	IVb	Liver, colon	3, Died of Disease
		74	F				X	3	0c	1	IVb	None	2, Died of Disease
		89	F				X	3	0c	1	IVb	Liver	1, Died of Disease
Adenosquamous/squamous cell carcinoma of the gallbladder	Chan KM, Yu MC, Lee WC, Jan YY, Chen MF	66	F	Cholelithiasis, abdominal pain, fever, jaundice, weight loss			X	3	1	1	IVb	Liver	3.2, Died of Disease
		62	F			X		3	1	0	III	Liver	18.4, Died of Disease
		72	F				X	4	1	0	IVa	Liver, duodenum, colon, bile duct	4.7, Died of Disease
		54	F				X	4	1	0	IVa	Liver, bile duct	9.3, Alive
		72	F				X	4	1	0	IVa	None	87.3, Died of Disease
		49	M				X	3	1	0	III	None	3.7, Died of Disease
		68	F			X		3	1	0	III	Liver	4.2, Died of Disease
		89	F					4	1	0	IVa	Liver, duodenum	0.8, Died of surgical mortality
		55	M			X		4	1	0	IVa	Liver, stomach	6.4, Died of Disease
		67	F			X		4	1	0	IVa	Liver, bile duct	1.5, Died of surgical mortality
		75	M				X	3	0	0	III	None	14.0, Died of Disease
		66	F				X	3	1	1	IVb	Liver	1.5, Died of Disease
Surgical resection of splenic metastasis from the adenosquamous gallbladder carcinoma: A case report	Utsumi M, Aoki H, Kunitomo T, Mushiake Y, Kanaya N, Yasuhara I, Arata T, Katsuda K, Tanakaya K, Takeuchi H	62	F	Abdominal pain	8.0 cm	X		4	1	1	IV	Liver, colon, spleen, diaphragm	Alive
A primary adenosquamous gallbladder carcinoma with sarcomatoid features	Qian X, Wu Y, Gao B, Wang W	51	F	Abdominal pain, anemia, abnormal LFTs	4.5 x 7.0 cm	X							5, Died of Disease
Long-term survival of a patient with advanced adenosquamous carcinoma of the gallbladder after radical resection	Fujita T, Fukuda K, Ohmura Y, Nishi H, Mano M, Komatsubara S, Doihara H, Shimizu N	72	F	Fatigue, weight loss, anorexia	4.5 cm	X		4	0	0	IVa	Stomach	60, Alive
Hepatopancreatoduodenectomy for squamous and adenosquamous carcinoma of the gallbladder	Miyazaki K, Tsutsumi N, Kitahara K, Mori M, Sasatomi E, Tokunaga O, Hisatsugu T	70	M			X		4	1b	0	IV	Liver, duodenum	6, Died of Disease
Adenosquamous carcinoma of the gallbladder: a clinicopathological, immunohistochemical and flow-cytometric study of 20 cases	Nishihara K, Nagai E, Izumi Y, Yamaguchi K, Tsuneyoshi M	68	F	Cholelithiasis	Mean: 6.3 cm Range: 3.8–10.6 cm	Radical resection performed in 10 out of 20 patients	Primary tumor resection performed in 10 out of 20 patients				Stage II: 3 Stage III: 11 Stage IV: 6	Cystic duct, peritoneum	3, Died of Disease
		52	F	Cholelithiasis								None	12, Died of Disease
		64	F									None	5, Died of Disease
		72	M									Liver	3, Died of Disease
		63	F	Cholelithiasis								None	6, Died of Disease
		73	F									Duodenum	1, Died of Disease
		63	F									LN	11, Died of Disease
		78	F	Cholelithiasis								Duodenum	7, Died of Disease
		66	F	Cholelithiasis								None	6, Died of Disease
		78	F									None	5, Died of Disease
		73	F	Cholelithiasis								Liver, colon	12, Died of Disease
		50	F									Liver	6, Died of Disease
		64	M									Liver	6, Died of Disease
		68	M	Cholelithiasis								None	56, Alive
		63	M									None	3, Died of Disease
		64	M									Liver, colon	3, Died of Disease
		71	F									None	19, Died of Disease
		76	M									None	3, Alive
		72	M	Cholelithiasis								Omentum	2, Alive
		60	M	Cholelithiasis								None	5, Died of Disease
Cholecystic adenosquamous carcinoma mimicking Mirizzi syndrome	Horio T, Ogata S, Sugiura Y, *et al.*	73	F	Cholelithiasis, Obstructive jaundice			X					Hepatoduodenal ligament	4, Died of Disease
Adenosquamous carcinoma of the gallbladder with tumor thrombus in left portal trunk	Iyomasa, S., Matsuzaki, Y., Hiei, K. *et al.*	73	M	Abdominal pain, palpable epigastric mass		X						Liver, L portal trunk	120, Alive
Ruptured adenosquamous cell carcinoma of the gallbladder: case report and review of literature	Rustagi T, Rai M, Menon M	74	F	Cholelithiasis, abdominal pain, weight loss, anorexia, fever, hepatomegaly	11 x 10 x 10 cm	X		3	1	0	III	Liver	Alive
A case of primary adenosquamous/squamous cell carcinoma of gallbladder directly invaded duodenum	Saito A, Noguchi Y, Doi C, Mukai K, Fukuzawa K, Yoshikawa T, Amano T, Kondo J, Ito T, Izutsu H	67	F			X						Liver, duodenum, pancreas, colon	4, Died of Disease
Adenosquamous carcinoma of gallbladder presenting as chronic cholecystitis with cholelithiasis- a rare entity	Mohan N, Agrawal R, Kumar P	45	F	Cholelithiasis, abdominal pain and distention, fever, SOB, constipation	9.5 × 5.5 × 3.5 cm		X						

Important prognostic factors of this disease are histologic grade and stage of the tumor. Because patients commonly present late, prognosis is often poor. In this population, 85% (59/69) presented at an advanced stage (defined as pT3 and greater OR Stage III and greater). The most involved adjacent structure in this population was the liver, which occurred in 53% (37/69) of patients. The overall 5-year survival rate has been estimated to be less than 5%. In this population, 60.8% (42/69) of patients died of their disease with a mean survival of 8.5 months prior to passing.

Treatment of this disease is difficult. Chemotherapy has shown little success, so surgery is often the gold standard. Two main approaches exist in the surgical treatment of gallbladder cancer: radical resection vs. resection of primary tumor alone; 52.9% (36/58) of this population underwent radical resection. It has been reported, however, that the overall survival rate is significantly better after radical resection when compared with primary resection of tumors that were incidentally found after a standard cholecystectomy.

## CONCLUSION

Gallbladder carcinoma is a rare cancer with the most common subtype being adenocarcinoma. Adenosquamous carcinoma, a much less common subtype, has been shown to have more aggressive biologic behavior than adenocarcinoma. This greater proliferative capacity often leads to tumor extension to adjacent structures such as the liver rather than nodal metastasis. There is conflicting evidence in the literature regarding the prognosis of adenosquamous carcinoma compared with adenocarcinoma, although both usually have a poor outcome. With little role for chemotherapy, surgery currently appears to be the gold standard. Due to the lack of available literature on this rare disease, more studies are needed to determine a more targeted approach of treatment.
